# Function and Regulation of MicroRNAs and Their Potential as Biomarkers in Paediatric Liver Disease

**DOI:** 10.3390/ijms17111795

**Published:** 2016-10-27

**Authors:** Diego A. Calvopina, Miranda A. Coleman, Peter J. Lewindon, Grant A. Ramm

**Affiliations:** 1 Hepatic Fibrosis Group, QIMR Berghofer Medical Research Institute, 300 Herston Rd, Herston, QLD 4006, Australia; Diego.Calvopina@qimrberghofer.edu.au (D.A.C.); Miranda.Coleman@qimrberghofer.edu.au (M.A.C.); Peter.Lewindon@health.qld.gov.au (P.J.L.); 2 Department of Gastroenterology and Hepatology, Lady Cilento Children′s Hospital, 501 Stanley St, South Brisbane, QLD 4101, Australia; 3 Faculty of Medicine and Biomedical Sciences, The University of Queensland, Brisbane, QLD 4006, Australia

**Keywords:** microRNA, children, chronic liver disease, circulatory miRNA

## Abstract

MicroRNAs (miRNAs) are short non-coding RNAs involved in biological and pathological processes of every cell type, including liver cells. Transcribed from specific genes, miRNA precursors are processed in the cytoplasm into mature miRNAs and as part of the RNA-induced silencing complex (RISC) complex binds to messenger RNA (mRNA) by imperfect complementarity. This leads to the regulation of gene expression at a post-transcriptional level. The function of a number of different miRNAs in fibrogenesis associated with the progression of chronic liver disease has recently been elucidated. Furthermore, miRNAs have been shown to be both disease-and tissue-specific and are stable in the circulation, which has led to increasing investigation on their utility as biomarkers for the diagnosis of chronic liver diseases, including those in children. Here, we review the current knowledge on the biogenesis of microRNA, the mechanisms of translational repression and the use of miRNA as circulatory biomarkers in chronic paediatric liver diseases including cystic fibrosis associated liver disease, biliary atresia and viral hepatitis B.

## 1. Introduction

In the US alone around 15,000 children are hospitalized for liver disease each year [1], however, the relative lack of epidemiological research studies in children masks the true prevalence of chronic liver disease which is likely underestimated. In fact, paediatric liver disease has an important impact on health care costs, leads to premature deaths and impacts quality of life in affected children.

Paediatric liver diseases are often diagnosed late, mainly due the lack of symptoms during early stages of the disease. The diagnosis is increasingly challenging when symptoms are non-specific such as loss of appetite, abdominal pain or fatigue. Liver biopsy remains the “gold standard” for diagnosis of liver disease and detection of hepatic fibrosis. However, liver biopsy is an invasive technique with potential for severe complication including bleeding, biliary peritonitis and pain. Moreover, liver biopsy is limited by sampling only a small part of the liver leading to sampling and diagnostic errors in liver disorders with a heterogeneous disease distribution. These issues highlight the need for non-invasive diagnostic methods to directly assess liver diseases and the degree of hepatic fibrosis. MicroRNAs (miRNAs) are considered as potential biomarkers for several chronic disorders due to their stability in the circulation, and are both disease- and tissue-specific, which makes them attractive circulatory biomarkers.

miRNAs are short interfering RNAs which catalytically silence gene expression at a post-transcriptional level. They constitute the most abundant class of endogenous, small, non-coding RNA with approximately 50,000 copies per cell in the liver [2]. Since Ambros′ discovery in 1993 [3], miRNAs have been extensively studied due to their role in RNA-induced silencing. In 2000, the miRNA let-7 was identified [4]. In contrast to previous miRNAs described, let-7 was shown to be widely conserved across different animal species [5]. This breakthrough discovery started an intensive search for novel miRNAs conserved over different species, including humans [2,6,7,8].

miRNAs are involved in the regulation of all biological and pathological processes in every cell type, including liver cells. Further, altered expression of miRNAs correlate with different liver aetiologies or are involved in the broader fibrogenic response to liver damage. The expression profiles of miRNAs also seem to be specific when compared between liver diseases of different aetiologies.

This review explores the current knowledge about the biogenesis of miRNAs, their regulation of gene expression, target identification and application as biomarkers in chronic paediatric liver disease.

## 2. Biogenesis of miRNAs

**Figure 1:**
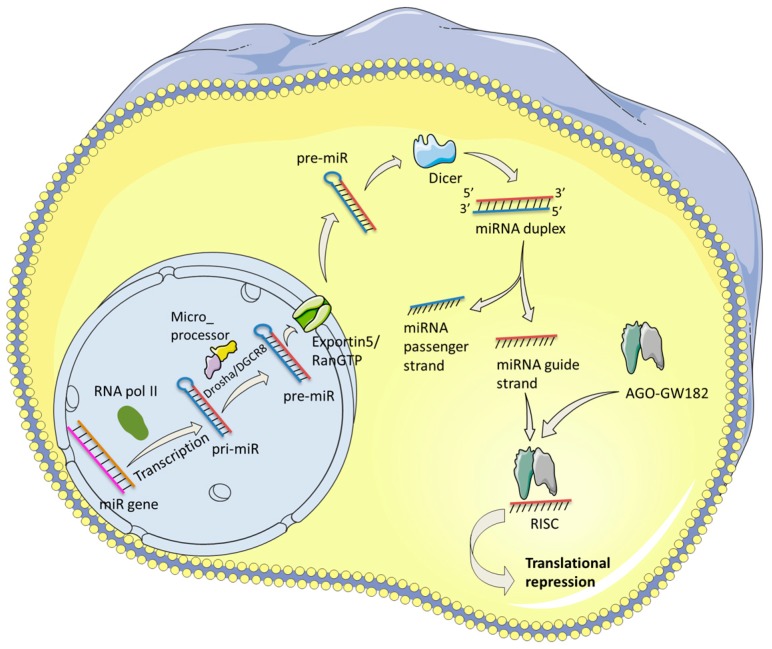
miRNA biogenesis. miRNAs are transcribed from miRNA genes by RNA pol II into pri-miRNA and cleaved by the microprocessor. The resulting pre-miRNA is exported to the cytoplasm by the exportin 5/RanGTP complex. Once in the cytoplasm, the endoribonuclease Dicer cleaves the pre-miRNA into a miRNA duplex. The mature miRNA is loaded into the AGO/GW182 complex forming the RISC complex, which mediates the translational repression. RNA pol II: RNA polymerase II; pri-miR: primary miRNA; pre-miRNA: precursor miRNA; AGO: argonaute; RISC: RNA-induced silencing complex.

Mature miRNAs are single stranded RNAs of about 17–24 nucleotides (nt) which interact with RNA-induced silencing complex (RISC) in the cytoplasm of eukaryotic cells [9]. The pathway leading to this regulatory interaction and the steps involved in biogenesis of miRNA are depicted in Figure 1. miRNAs are encoded by specific genes transcribed by RNA Pol II into polyadenylated and capped stem-loop transcripts termed primary miRNAs (pri-miRNAs) [10,11]. Most miRNA genes encode a single miRNA, however, some are encoded in clusters and can include up to six miRNAs with a similar sequence [12]. A minority of miRNAs are located in introns of coding genes which form miRNA precursors during splicing [13]. miRNA biogenesis is regulated at a transcriptional and post-transcriptional level and single nucleotide polymorphisms (SNPs) in miRNA genes can modulate activity and function [14].

pri-miRNA undergoes a process of maturation in the nucleus, mediated by a complex called Microprocessor. The Microprocessor is formed by RNase III endonuclease Drosha and RNA-binding protein DGCR8 [15,16]. Drosha cuts the stem loop of the pri-miRNA, releasing a hairpin shaped RNA of 60–70 nt with a two nucleotide 3′ overhang named precursor miRNA (pre-miRNA) [17].

Once Drosha has processed pre-miRNA, it is exported to the cytoplasm by a complex composed of exportin 5 and the GTP-binding nuclear protein RanGTP [18,19]. In the cytoplasm, pre-miRNA is processed by the RNase III endonuclease called Dicer [20,21]. Dicer recognizes the 5′ phosphate and 3′ overhang at the base of the stem loop and cuts both RNA strands, liberating a mature miRNA with 5′ phosphate and two nucleotide 3′ overhang on each end of the double stranded RNA [22,23]. The mature miRNA processed by Dicer consists of a guide strand, which is antisense to the target sense strand on mRNA, and an unstable passenger strand.

The recently formed mature miRNA is loaded onto a protein called Argonaute (AGO) which is a family of four members [24]. This interaction forms an effector complex known as RNA-induced silencing complex (RISC) [25,26]. AGO proteins present a PAZ domain in the N-terminal lobe which binds to single stranded and duplex RNA [27,28].

Assembly of RISC involves two steps: the binding of miRNA duplex and its unwinding [29]. In humans there is no strict RNA sorting system as in other species such as *Drosophila* [30]. RNA binds to any of the four AGO proteins with preference of small RNA duplexes with central mismatches between nucleotides in position 8–11 [31,32]. Once the RNA duplex is bound to an AGO protein, the passenger strand is removed to generate the mature and functional RISC complex [25]. This process is mediated by AGO2 which has helicase and endonuclease activity [33]. The guide strand presents mismatches at positions 2–8 and 12–15 nt that promote the unwinding of the duplex [31,34].

## 3. Mechanisms of Translational Repression

**Figure 2:**
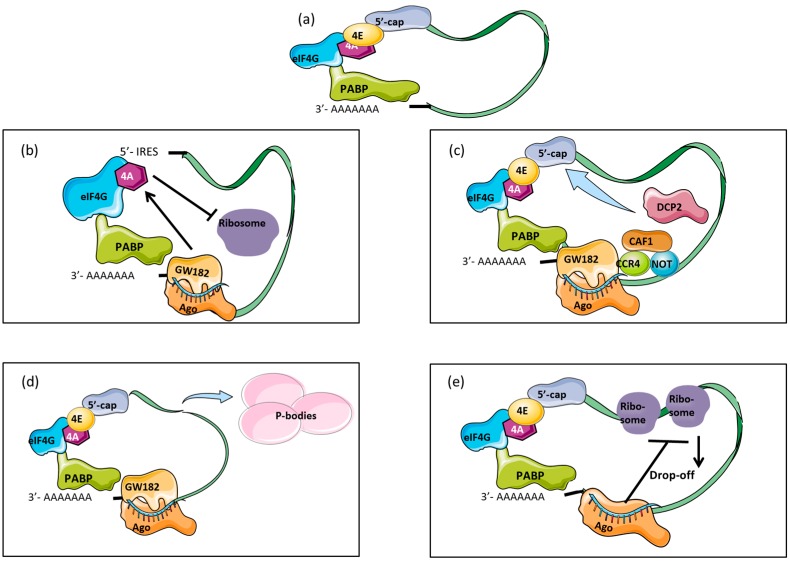
miRNA methods of translational repression. (**a**) mRNA is effectively translated when it possesses a 5′-cap and 3′-poly(A) tail. At the initiation of translation, PABP associates with eIF4G which interacts with the 5′-cap structure forming a circular mRNA protected from degradation; (**b**) In the cap-independent mechanism, IRES binds to EIF4G without the need of EIF4E. After miRNA-mRNA binding, translational repression is mediated by EIF4A on the 5′ UTR preventing the action of ribosomes; (**c**) During the 5′–3′ decay pathway, GW182 as part of RISC, interacts with PABP and recruits the CAF1-CCR4-NOT complex which deadenylates mRNA. After deadenylation, mRNA is decapped by DCP2 and finally degraded; (**d**) mRNA degradation associated to p-bodies occurs after binding of RISC containing the miRNA. mRNA is sequestered to cytoplasmic P-bodies, which are centres of mRNA degradation containing key proteins necessary for translational repression; (**e**) In the ribosome drop-off model, miRNA associated with Argonaute binds to the mRNA target and has a distanced effect on translating ribosomes at multiple sites causing the ribosomes to drop-off. eIF4: eukaryotic translation-initiation factor 4; PABP: cytoplasmic poly(A) binding protein; IRES: internal ribosome entry site; DCP2: decapping enzyme 2.

Efficient translation occurs when mRNAs possess a 5′-cap (5′-7-methylguanine or m7GpppN) and a 3′-poly(A) tail. During translation initiation, the cytoplasmic poly(A) binding protein (PABPC) associates with the poly(A) tail and acts together with the eukaryotic translation-initiation factor 4G (eIF4G). At the same time, eIF4G interacts with the 5′-cap structure, forming a circular mRNA that is protected from degradation and can be effectively translated [35,36] (Figure 2a). miRNAs interfere with the function and interaction of PABPC and eIF4G, inhibiting translation at the initial stage [37,38]. However, there are several mechanisms by which miRNAs may cause mRNA repression including a cap-independent mechanism, 5′–3′ mRNA decay pathway, formation of pseudo-polysomes and ribosome drop-off model (Figure 2).

In cap-independent mRNA, miRNAs can silence translation through an internal ribosome entry site (IRES) [38,39] (Figure 2b). GW182 protein is part of the RISC complex that mediates translational repression. Once the target is identified by the miRNA within the RISC complex, GW182 interacts with PABPC (Figure 2c). The activated GW182 recruits the CAF1-CCR4-NOT deadenylase complex which deadenylates the mRNA [40,41,42]. After deadenylation, the mRNA is decapped by decapping enzyme DCP2 [43]. The deadenylated and decapped mRNA is then degraded by the cytoplasmic 5′–3′ exonuclease XRN1 [44]. This process is known as the 5′–3′ mRNA decay pathway [45] (Figure 2c).

Another model of repression has been described in *Drosophila* in which pseudo-polysomes are assembled from large miRNAs and mRNA forming a dense messenger ribonucleoprotein (mRNPs) complex heavier than the 80 s ribosome (Figure 2d) [46]. Pseudo-polysomes interact with structures called P-bodies that reside in the cytoplasm of eukaryotic cells and have been linked to mRNA degradation [47,48]. During the ribosome drop-off model, the interaction between ribosomes and mRNA is released when miRNA from the RISC complex binds the 3′UTR of the mRNA [38] (Figure 2e). Evidence for the existence of this model has been shown in polypeptides that undergo translation and are rapidly degraded under the regulation of specific miRNAs in contrast to inhibition at initial stages [49].

## 4. miRNA Target Identification

For all of the aforementioned translational repression models, identification of the target mRNA by the miRNA within the RISC complex is essential. Watson-Crick base pairing of the nucleotides on the 5′ end of the miRNA and the target mRNA take place during this interaction [50,51,52]. The region in which mRNA and miRNA hybridization occurs is termed the “seed” sequence [50,53] and consists of a minimum of six base pairs (bp) which correspond to nucleotides 2–7 from the 5′ end of the miRNA (3′UTR of the mRNA). The seed sequence can be up to 8 bp in length, based on the homology at position eight or the presence of an adenine (A) at nucleotide position one of the target mRNA [53].

However, seed matching alone is not enough to identify validated targets [54]. The context in which seed matching occurs plays an important role in determining miRNA targets in the canonical 3′UTR or different segments of the mRNA. Grimson et al. [54] described the characteristics of the most common miRNA targets (Table 1). These parameters have been implemented in computational tools and algorithms that predict miRNA targets.

miRNA downstream targets have been the focus of many animal studies designed to elucidate their involvement in fibrogenesis associated with chronic liver disease (reviewed elsewhere [55]). Only recently with interest in miRNAs as circulatory biomarkers have miRNAs been correlated with fibrosis in chronic liver diseases of varying aetiologies such as alcoholic liver disease (ALD) [56,57], non-alcoholic fatty liver disease (NAFLD) [58,59,60,61], chronic hepatitis C [58,62,63], chronic hepatitis B [64,65] or drug induced liver injury [66] to name a few. Most of these studies assessing the circulatory miRNA signature have been conducted in adult chronic liver diseases with a paucity of information available in the paediatric setting.

## 5. Circulatory miRNAs as Biomarkers of Disease

miRNAs finely adjust rather than completely repress gene expression [67]. It is estimated that most genes expressed in mammals [68] are to some extent regulated by the 2588 mature miRNAs identified to date [69]. Many biological pathways are regulated by miRNAs including cellular proliferation, differentiation, apoptosis, cell cycle regulation and development. Furthermore, the roles of miRNAs have been described in several diseases, including cancers [70,71,72,73], coronary diseases [74,75,76], autoimmune diseases [77,78,79] or viral infections such as viral hepatitis [80,81,82,83]. miRNAs predominantly exist intracellularly, however, it is possible to find miRNAs in extracellular environments such as in serum, plasma, semen, cerebrospinal fluid and urine [84,85,86,87,88,89,90,91]. Two hypotheses are proposed to explain the origin of circulatory miRNAs. The first suggests cells evolved to selectively release miRNAs via paracrine or endocrine routes to mediate cell–cell signalling [92,93]. The second proposes that miRNAs are released in a non-selective manner after cell death, correlating with increased levels of miRNAs in blood after toxicity in certain organs [57,94,95]. Circulatory miRNAs are relatively stable, especially compared with extracellular RNAs. miRNAs are packed in vesicles or in association with RNA binding proteins to prevent digestion by ribonucleases (RNases) [96].

The transfer of miRNA packed into extracellular vesicles (EVs) strengthens the hypothesis of direct cell–cell contact and selective release as a mechanism of intercellular communication. This enables miRNAs to regulate cell function in a paracrine manner or through the circulation and in different body fluids to reach cells in distal organs [97]. The term exosome was first used in 1983 referring to EVs [98]. Exosomes are formed in endosomal compartments called multivesicular endosomes (MVEs) by budding of the internal vesicle that contains proteins, mRNA and miRNA from the cell cytoplasm. Exosomes are actively secreted by fusion of the MVE with the plasma membrane (exosome biogenesis reviewed in [99]).

The different steps of MVE formation are highly regulated and include segregation of cargo, delimitation of the endosome membrane and the budding of vesicles in the endosome. The endosomal sorting complex required for transport (ESCRT) recognizes ubiquinated proteins and promotes their internalization into the MVEs during its formation [100]. Exosomes have a mean size of 40–100 nm [101]. To purify exosomes, a series of centrifugation and ultracentrifugation steps are used [102]. However, other EVs can be isolated along with exosomes and establishing methods for exosome purification based on size, density, morphology and membrane and protein composition remains a challenge [103].

Increasing interest in exosomes as disease biomarkers began a decade ago when Valadi et al. [93] reported that exosomes isolated from cultured cells contained both miRNA and mRNA. Furthermore, Valadi et al. showed that the mRNA was effectively and functional translated once the exosome was internalized in the target cell. This study was followed by reports of miRNA in exosomes isolated from different body fluids including blood and saliva [104,105,106]. Studies have revealed a distinctive miRNA profile in exosomes when compared to the miRNA profile of the cells from which the exosomes were formed [107,108]. This suggests that miRNAs are selectively incorporated into exosomes and not as a consequence of apoptosis or degradation of cellular components.

How miRNAs are sorted into exosomes remains unclear. Specific sequence motifs shared by miRNAs in EVs have been suggested to control their sorting into exosomes [109]. Moreover, it has been proposed that the heterogeneous nuclear ribonucleoprotein A2B1 (hnRNPA2B1) recognizes these specific miRNA motifs and controls their loading into exosomes [110]. Additionally, the presence of GW182 and AGO2 proteins in exosomes [111], both key molecules necessary for the RISC formation, suggests their potential role in exosome miRNA sorting although the precise mechanisms responsible remain to be elucidated.

Evidence suggests that released exosomes interact with specific target cells [112,113]. Target cell specificity depends on distinctive molecules including integrins [114], tetraspanin complexes [115] and others such as galactin-5 and galectin-9 [116]. After binding with the target cell, exosomes fuse with the plasma membrane and release their contents into the cytoplasm. Alternatively, exosomes have been shown to be internalized via endocytosis where they fuse with the endosomal membrane releasing their contents and/or be targeted for lysosomal degradation.

Regardless of the origin of circulatory miRNAs, their presence in readily accessible body fluids clearly make miRNAs attractive biomarker candidates. The ideal biomarker should meet stringent criteria, such as being disease-specific, detected in a non-invasive manner, an indicator of disease at an early stage, or responsive through the progression of the disease or treatment. miRNAs are indeed disease- and tissue-specific, stable in circulation and capable of distinguishing between healthy and diseased individuals, further strengthening the attraction of circulating miRNAs as biomarkers [117].

## 6. Methodological Challenges in the Study of Circulatory miRNAs

Pre-analytical variation can affect the quantification of miRNAs. During sample collection it is essential to remove cellular components such as erythrocytes, leukocytes and platelets from blood. In particular, erythrocytes express high levels of miR-451 and miR-16, which correlates with haemolysis in plasma samples [118,119]. Heparin, a traditional anticoagulant used in plasma, shows a dose dependent inhibitory effect by binding to calcium and magnesium used in PCR reactions [120,121]. Although miRNAs are resilient in serum or plasma, repeated freeze-thaw cycles decreases detectable miRNA levels and should be avoided [122].

One major challenge is the relatively low abundance of miRNA in plasma and serum along with high levels of proteins and lipids which can interfere with the isolation process. Originally, TRIzol was used as an effective method for small RNA isolation, however, phenol contamination and difficulty with pellet resuspension regularly interfered with the process. These problems have been partially solved by method modifications including small RNA binding columns common to multiple commercial kits.

Traditional RNA quantification methods, such as spectrophotometer or capillary electrophoresis, are unable to assess size or quality of the isolated miRNA accurately, due to the low yield and limits of detection. Quantitative reverse transcription-PCR (qRT-PCR) is currently considered the gold standard method for assessing isolated miRNA [119,123].

## 7. Circulatory miRNAs as Biomarkers in Paediatric Liver Disease

Several studies have explored the potential targets and thus the mechanistic role of specific miRNAs in chronic liver diseases, including those affecting children (Table 2). These studies have used liver tissue from children or animal models to identify miRNA expression profiles in the context of the specific disease. Once miRNAs of interest had been selected, liver and biliary cell lines were used to identify potential gene targets and their effect on protein synthesis, mRNA expression or the role they play in cellular biological pathways including proliferation, migration or apoptosis. These studies not only show the importance of gene regulation through miRNA in liver disease, but also the complex mechanisms that drive fibrosis in which miRNAs also play an important role. For instance, miR-122 has been the subject of extensive investigation in liver disease. miR-122 knockdown experiments in mice demonstrated important hepatopathological effects as mice aged including hepatocyte proliferation, imbalance in cellular differentiation and ductular reaction [124]. Similarly, multiple studies have shown increased serum levels of miR-122 corresponding to different liver aetiologies, suggesting that upregulation of miR-122 in serum may be a global marker of liver injury rather than disease-specific [57,58,125,126]. These evident roles for miRNAs in physiological and pathological pathways and their reflection in the circulation could be utilised for the non-invasive detection of chronic liver diseases in children.

In recent years, there has been significant interest in investigating the potential for circulatory miRNAs to act as biomarkers of liver disease in adults; however, few studies have been conducted in paediatric liver disease. Those few studies that have examined the potential role of circulating miRNAs in the detection of liver disease have focussed on children with either cystic fibrosis-associated liver disease (CFLD), biliary atresia (BA) or viral hepatitis B.

### 7.1. Cystic Fibrosis Liver Disease (CFLD)

CFLD is a major complication of cystic fibrosis (CF), responsible for up to 5% of mortality [142]. Sera from children diagnosed with CFLD, cystic fibrosis but no liver disease (CFnoLD) and healthy control children (control) were collected to identify a circulating miRNA signature in CF children (Table 3) [143]. Initially 84 miRNAs were evaluated using a PCR array and validated by qRT-PCR. Analysis revealed upregulation of miR-122 in the CFLD group compared to both CFnoLD and control groups. Of interest this study demonstrated an elevation in serum miR-21 and miR-25 in CFnoLD compared to both CFLD and control groups. Therefore, a panel consisting of miR122, miR-21 and miR-25 showed the potential for early diagnosis of CFLD in clinical settings.

**Figure 3:**
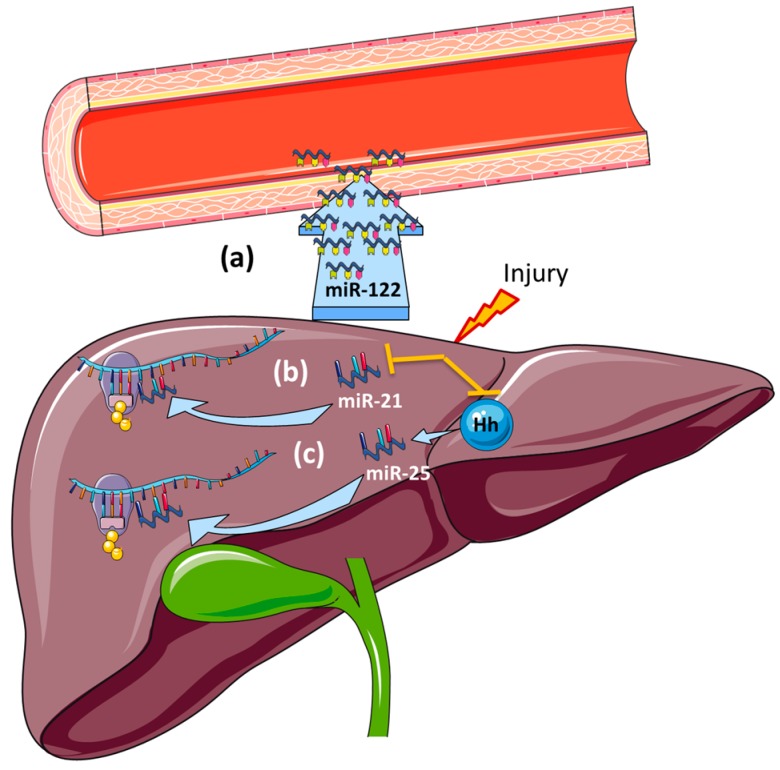
Proposed role of miRNA biomarkers in CFLD. (**a**) After liver injury, hepatocyte death causes the release of miR-122 into the bloodstream; (**b**) miRNA-21 has a role in the fibrogenic process by targeting genes involved in cell death and ECM remodeling; (**c**) Hedgehog (Hh) signalling mediates the expression of miRNA-25 which targets apoptotic genes during liver injury. Hh: Hedgehog signalling.

CFLD is believed to arise as a result of impaired function of the cystic fibrosis transmembrane regulator (CFTR) in cholangiocytes, which results in decreased bile flow and blockage of bile ducts. The retention of toxic bile acids [150] within bile ducts and the liver results in hepatocellular injury and activation of hepatic stellate cells (HSC), the principal collagen-producing cell in the liver. This leads to hepatic fibrosis and ultimately, in most, a variable severity of biliary fibrosis progressing to cirrhosis in up to 10% of children [151]. It is possible that the increased level of serum miR-122 detected in CFLD [143] may be caused by hepatic cell death (Figure 3a), although this requires further investigation. The same study also showed a positive correlation between serum miR-122 and both aspartate amino transferase (AST) and alanine amino transferase (ALT) suggesting a reflection of the liver integrity during disease progression [143].

miR-21 has been proposed to target genes involved in cell death and extracellular remodelling in different types of cancer including squamous cell carcinoma, as well as both hepatocellular and cholangiocarcinoma [152,153,154]. Others have shown that the induction of oxidative stress by cytotoxic bile acids reduces the expression of miR-21 in hepatocytes facilitating apoptosis [155]. This suggests that miR-21 may play an important role in liver homeostasis and regeneration [156] (Figure 3b). Moreover, a recent study in non-alcoholic steatohepatitis (NASH) revealed that after oxidative stress, miR-21 elicits fibrogenesis as part of the regeneration process [140].

microRNA-25 has been implicated in the regulation of apoptosis acting as an anti-apoptotic agent in cholangiocarcinoma [157]. It has been previously shown that activation of Hedgehog signalling, which plays a role in the fibrogenic process are associated with the overexpression of the miR-106b-25 cluster, which miR-25 is part of [158]. DR4, a component of the TRAIL death signalling pathway, is targeted by miR-25 preventing cell apoptosis [157] (Figure 3c). Thus, we propose a hypothetical schema for the possible role of these miRNAs in CFLD and their potential to act as biomarkers in CFLD (see Figure 3). However, further functional studies are required in CFLD to demonstrate a disease specific role for miR-21 and miR-25.

### 7.2. Biliary Atresia

Biliary atresia (BA) is a neonatal liver disease characterized by inflammation and fibrosis of the extrahepatic biliary ducts, leading to cholestasis and biliary cirrhosis [159]. In order to restore bile flow, the only treatment is a Kasai procedure (hepatoportoenterostomy) [159]. However, more than 70% of patients with a successful Kasai procedure will require a liver transplant during childhood due to ongoing sclerosis of the bile duct [160].

As in the study conducted in CFLD, serum miRNA profiles have been evaluated using PCR arrays in children with BA versus a cholestatic control group including children diagnosed with non-BA cholestatic diseases such as Alagille syndrome or choledocholithiasis (Table 3) [144]. Eleven miRNAs were found to be differentially expressed between the two groups and nine confirmed by qRT-PCR in independent cohorts. miR-200a, miR-200b and miR-429 (members of the miR-200 cluster) were significantly upregulated in BA patients compared to the cholestatic control group. Receiver operating characteristic (ROC) analysis showed the diagnostic utility of miR-200a, miR-200b and miR-429 by correctly diagnosing up to 85% of patient samples. However, it remains to be demonstrated whether a combination of these microRNAs in a diagnostic panel could improve accuracy.

Similarly, Dong et al. [145] performed a study in 45 BA children and 20 non-BA cholestatic children as controls. Serum from four BA children and four non-BA cholestatic controls was used for the initial microarray analysis identifying 13 differentially expressed miRNAs. Eight miRNAs were selected for validation based on predicted enriched functions and pathways of targeted genes. The validation was performed by qRT-PCR in 10 BA children and 10 non-BA cholestatic controls revealing a decreased expression of miR-4429 and an increased expression of miR-92a-3p, miR-4689 and miR-150-3p in the BA group. In addition, a diagnostic utility analysis was performed by qRT-PCR in 35 BA children and 20 non-BA cholestatic controls. The analysis showed a significant downregulation of miR-4429 and upregulation of miR-4689 in the BA group while the remaining miRNAs were not significantly differentially expressed at this stage. The ROC curve analysis results in an area under the curve (AUC) of 0.789 and 0.722 for miR-4429 and miR-4689, respectively, which according to the authors suggests a promising diagnostic performance. Interestingly, the miRNAs reported by Zhan et al. [144] were not differentially expressed in the Dong et al. study which might be due to small sample size used during the microarray analysis (*n* = 4/group) or differences in sample cohorts selected. These recurrent differences between reported miRNA expression profiles stress the need for standardized methodological approaches and study designs, including prospective validation in large scale studies.

In BA, fibro-obliteration of the extrahepatic biliary tree results in significantly decreased or absent bile flow, leading to cholangiocyte and hepatocyte injury. Hence, an elevation of serum miR-122 might have been expected in BA patients as a marker of liver injury; however, this was not observed in either study. This may be due to the characteristics of the study groups, i.e., the comparator groups in each study were liver disease patients. If compared to a healthy control group, a differential miR-122 expression might have been detected.

In fact, a recent study compared plasma miRNA profiles in children with BA [146] to healthy controls. The study consisted in of an initial “discovery” phase with nine BA patients and nine healthy controls pooled in groups of three. cDNA libraries were constructed from each group and miRNA profiles compared by next-generation sequencing (NGS). From this initial discovery phase, 15 miRNAs were found to be differentially expressed, seven upregulated (miR-200a-3p, miR-574-5p, miR-194-5p. miR-432-5p, miR-122-5p, miR-100-5p and let-7c-5p) and eight downregulated (miR-10b-5p, miR-140-3p, miR-26a-5p, miR-126-3p, miR-744-5p, miR-370-3p, miR-142-3p and miR-23a-3p) in BA patients compared to healthy controls (Table 3). A second phase performed in 44 BA patients, 20 non-BA cholestatic disease patients and 20 healthy controls was used to validate the NGS findings. Based on the high read counts obtained during NGS (≥5000) six miRNAs (miR-122-5p, miR-100-sp, miR-140-3p, miR-10b-5p, miR-26a-5p and miR-126-3p) were chosen for validation. The validation phase showed an increase in the levels of miR-122-5p and miR-100-5p and decrease of miR-140-3p and 126-3p in BA patients compared to controls. In addition, BA patients showed a significant decrease of miR-140-3p when compared to cholestatic patients. ROC curve analysis was used to determine the efficacy of miR-140-3p to distinguish BA patients from non-BA patients (controls and other cholestatic disorders) showing a sensitivity and specificity of 66.7% and 79.1% respectively. While, decreased miR-140-3p was reported in this study as a potential biomarker to identify BA, there is a lack of clinical accuracy. The inclusion of miR-140-3p as part of an expanded panel of different miRNAs may improve diagnostic utility.

Peng et al. [146] describe an elevated expression of miR-200a which forms part of the miR-200 cluster, confirming the finding previously reported by Zhan et al. [144] in serum samples. As expected, an overexpression of miR-122-5p in plasma from BA patients was found when compared to healthy controls but not when compared to other cholestatic diseases, which suggest that miR-122 is in fact a marker of liver injury. Potential shortcomings of this study include the use of small (pooled) sample size in the NGS discovery phase to identify BA biomarkers. In addition, the decision to choose only miRNAs with read counts over 5000 could have negatively influenced the discovery of a more reliable miRNA diagnostic panel.

**Figure 4:**
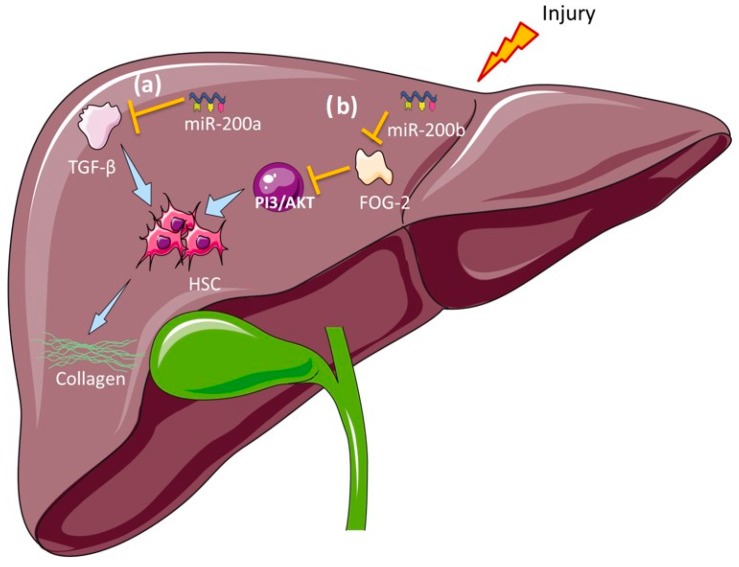
Proposed role of miRNA biomarkers in BA. (**a**) miRNA-200a is involved in the inhibition of TGF-β induced HSC activation and collagen synthesis, suggesting an anti-fibrotic role; (**b**) miRNA-200b enhances the PI3K/Akt signalling by targeting FOG2, which activates HSCs and collagen synthesis. TGF-β: transforming growth factor β; HSC: hepatic stellate cells; FOG2: friend of GATA protein 2.

Hepatic stellate cells (HSC) play an important role in the liver regeneration process and are responsible for fibrotic tissue synthesis in BA [161]. HSC can be activated by cytokines such as transforming growth factor β (TGF-β) [151], which acts as a profibrogenic mediator. miR-200a has been involved in epithelial to mesenchymal transition (EMT) and cell migration mediated by TGF β-1 signalling in breast cancer [162], and in cardiac [163], renal [164] and pulmonary fibrosis [165]. It is also involved in the inhibition of TGF-β by targeting ZEB1 and ZEB2 during renal injuries (Figure 4a) [166,167]. This evidence suggests an anti-fibrotic role for miR-200a in reversing fibrogenic activity [167]. We proposed that the elevation of miR-200a in serum might originate from the destruction of cells during the progression of the disease, which correlates with the decrease of miR-200a observed in BA liver tissue from mice models [127].

As part of the miR-200 cluster, miR-200b and miR-429 have also been associated with EMT during pulmonary fibrosis, intestinal fibrosis and breast cancer [165,168,169]. miR-200b has been shown to correlate with liver fibrosis progression in BA patients by targeting FOG2, a known inhibitor of PI3K/Akt signalling [128] which is important for the activation of HSC (Figure 4b). Thus, a role for these miRNAs as BA biomarkers is proposed, with potential mechanism of action in BA liver depicted in Figure 4. However, once again, further mechanistic studies are required to validate these possible pathways of miRNA regulation of hepatic fibrogenesis in BA.

### 7.3. Viral Hepatitis B

Chronic hepatitis B (CHB) is a global health burden that according to the World Health Organization (WHO) affects an estimated 240 million people worldwide [170]. Children can be infected during birth or at early infancy, with infections persisting in 90% of cases and around 5% developing chronic disease [171]. CHB is defined as positive for surface antigen of hepatitis B virus (HBsAg) for over six months. Liver damage in viral hepatitis is caused by immune responses to cells infected with the virus which over the course of CHB presents three characteristic stages: immune-tolerant, immune-active and immune-inactive [172]. Immunological phases are primarily characterised by the persistence of hepatitis Be antigen (HBeAg), high levels of hepatitis B virus and ALT. Children in the immune inactive stage have a lower risk of liver disease progression, however, hepatitis B virus reactivation may occur [173]. Winther et al. compared plasma miRNA levels in children with HBeAg+ and HBeAg− versus healthy controls (Table 3) [147]. Differentially expressed miRNAs were assessed by PCR arrays, identifying the upregulation of 16 miRNAs (miRNA-99a, miRNA-100, miRNA-122-5p, miRNA-122-3p, miRNA-125b, miRNA-192-5p, miRNA-192-3p, miRNA-193b, miRNA-194, miRNA-215, miRNA-356, miRNA-455-5p, miRNA-455-3p, miRNA-483-3p, miRNA-885-5p and miRNA-1247) for which expression was highest in the HBeAg+ group, lower in HBeAg− group and lowest in the healthy control group. A gene set enrichment analysis showed these miRNAs are involved in insulin, MAPK or WNT signalling pathways.

A follow up study (Table 3) [148] focused on the association of these 16 miRNAs with HBsAg over the course of CHB. Children were classified according to their immunological phase as immune-tolerant, immune-active and immune-inactive. While miR-455-3p and miR-1247-5p were not assessed due to technical issues, the remaining 14 miRNAs were all highly expressed in the immune-tolerant group, lower in immune-active group and lowest in immune-inactive group. Interestingly, levels of expression of HBsAg followed the same pattern among the three groups, showing a strong correlation between plasma miRNA levels, HBsAg and HBV DNA.

In a more recent study, Winther et al. identified liver specific target genes in plasma of HBeAg+ children (Table 3) [149]. Using the raw data from the original study [147], plasma miRNA expression was re-analyzed from HBeAg+, HBeAg− and the healthy control groups. Thirty-two miRNAs were shown to be significantly differentially expressed between HBeAg+ and HBeAg− children. In addition to the 14 miRNAs described in the previous studies [147,148], nine upregulated and nine downregulated miRNAs were identified.

Identification of liver specific miRNA targets of the 32 miRNAs was performed using CLIP-seq overlap. Out of these 32 miRNAs, 16 miRNAs were found to be liver specific. Furthermore, these 16 miRNAs were found to target 16 genes, with each miRNA targeting multiple genes. The identified target genes were: *ACADSB*, *ARIDIA*, *BTG3*, *CEBP6*, *CPOX*, *E2F1*, *FRAT2*, *GABBR1*, *GATA6*, *HOXA9*, *LEF1*, *MAZ*, *PAPD5*, *SF1*, *SMAD4* and *ZXDB*.

From the 16 miRNAs analysed, six were previously validated [147,148], the remaining were validated by qRT-PCR. Validation confirmed miR-28-5p, miR-30a-5p, miR-30e-3p, miR-378a-3p, miR-574-3p and let-7c to be upregulated and miR-654-3p to be downregulated in HBeAg+ compared to HBeAg− groups. Comparing the HBeAg− and healthy control groups, only miR-28-5p and miR-30a-5p were upregulated, whereas miR-654-3p showed no statistically significant difference.

These results suggest a correlation between the miRNAs described above, HBV DNA and HBeAg, thus implying a role in viral replication. The role of the aforementioned miRNAs during the progression of CHB was clear by studying their relationship with the different immunological phases. Winther et al. proposed that over time immunological control is possible with plasma miRNA levels as they inversely correlated to immunological control. This suggests that a panel of miRNAs could be used to identify the immunological status of patients. From the above identified miRNA target genes the majority have been described as regulators or suppressors of carcinogenesis or HBV replication [174,175,176,177,178]. This, once more, highlights the role of microRNA during the course of infection and liver damage; however, further studies are required to clarify the mechanisms involved.

Interestingly, some of the differentially expressed plasma miRNAs found by Winther et al. have also been reported in circulatory miRNA profiling in an adult population. Brunetto et al. [65] showed overexpression of serum miR-122-5p, miR-99a-5p and miR-192-5p between inactive HBsAg carriers and CHB patients. Furthermore, this miRNA expression was correlated to the levels of circulatory HBsAg particles. These observations in adults are consistent with the one reported by Winther et al. [148] comparing levels of plasma miRNAs in children with CHB considering HBsAg levels. Likewise, Ninomiya et al. [179] reported upregulation of serum miR-100-5p, miR-125b-5p, miR-193b-3p, miR-194-3p, miR-30a-3p, miR-30c-2-3p, miR-3591-5p, miR-4709-3p, miR-574-3p and miR-99a-5p of CHB adult patients compared to healthy controls. Overall, seven of these miRNAs including miR-99a, miR-100-5p, miR-194, miR-30a, miR-125b and miR-574, are consistently reported to be upregulated in CHB in both serum from adults [179] and plasma from children [147,148].

## 8. Conclusions

The true prevalence and morbidity of paediatric chronic liver disease is unknown, however, every year thousands of children are hospitalized for liver diseases of varying aetiologies. Ultimately, these diseases have a direct impact on quality of life, with significant associated costs to the health care system. In addition, these paediatric conditions are a precursor of chronic liver pathologies in adult life [180].

There is a clear need for reliable, non-invasive methods to identify liver disease at an early age, especially where timely intervention is critical for improved patient outcomes. Our understanding of miRNAs and their role in different liver pathologies is increasing rapidly. During the past two decades, 2588 mature miRNAs have been described in humans [69], miRNA mechanisms of translational repression [181] and target identification [182] have also been widely studied. Yet, microRNA involvement in liver disease, especially in paediatrics, needs further investigation and elucidation.

A key feature of miRNAs is their stability in serum and plasma due to their association with Ago2 [183], exosomes [93], apoptotic bodies [184] or HDL [185]. This robust stability, together with demonstrated organ- and disease-specific expression, enable miRNAs to act as potential non-invasive biomarkers for the detection and diagnosis of liver disease, and the assessment of hepatic fibrotic stage providing the ability to monitor the development of cirrhosis. Over the past ten years numerous studies, mostly conducted in animal models or in human tissue samples, have demonstrated a disease-specific link between specific miRNAs and chronic liver disease. The increasing literature exploring the role of miRNAs in liver has expanded our knowledge about the mechanisms that drive physiological and pathological hepatic functions. It is now known that miR-122 accounts for over half of the miRNAs expressed in liver followed by miR-192 and miR-199a/b-3p (52%, 17% and 5%, respectively) [186]. Along with this increased knowledge of hepatic miRNA function, there has been a natural exploration of their potential to act as both circulating biomarkers of disease status and indeed as novel therapeutics. However, a significant majority of these studies have been performed in adult chronic liver disease (reviewed elsewhere [55]), especially in the assessment of circulatory miRNA signatures. Considering the multiple pathologies causing paediatric liver disease, only cystic fibrosis associated liver disease, biliary atresia and chronic hepatitis B, have been studied to determine the utility of circulatory miRNAs as biomarkers of hepatic disease. Further paediatric studies are needed especially in children with NAFLD, as with an estimated prevalence of 7.6% in the general population and 34.2% in obese children [187], NAFLD is currently the most common cause of paediatric liver disease [188]. Indeed, there are a large number of additional paediatric liver diseases that may benefit from the use of miRNAs as biomarkers for differential diagnosis, determining prognosis and potentially to elucidate disease mechanism, including such conditions as Crigler-Najjar syndrome, Gilbert syndrome, Wilson disease, hepatoblastoma, angiosarcoma, infantile hepatic haemangioma, allagile syndrome, A-1 antitrypsin disorder and autoimmune hepatitis.

In order to complete the translation of these studies into the clinical setting, some challenges need to be addressed. The establishment of standardized methodological design for low yield isolation from biologic fluids, accurate quantification and normalization will greatly benefit reproducibility and translation into the clinic. As our understanding of miRNAs increases, new opportunities will arise in their use not only as diagnostic tools but also as potential therapeutic treatments for both paediatric chronic liver disease and liver cancer.

## Figures and Tables

**Table 1:** Parameters for the identification of miRNA targets.

Parameters
Closely spaced miRNAs often act synergistically Watson-Crick pairing at nucleotide 12–17nt in addition to seed match enhanced miRNA targeting
Effective targets reside within locally AU-rich context
Effective targets reside in 3′ UTR strand but not close to stop codon
Effective sites preferentially reside near both ends of the 3′ UTR

**Table 2:** Functional miRNA studies in paediatric liver diseases.

Disease	Sample Type	Sample Source	Upregulated	Downregulated	References
Billiary atresia (BA)	Extrahepatic bile ducts	mice	–	miR-30b/c miR-133a/b miR-195 miR-200a miR-320 miR-365	Bessho et al. [127]
	LX2 (cell line) Liver tissue	human	miR-200b	–	Xiao et al. [128]
	Liver tissue	human	miR-21	–	Shen et al. [129]
	Liver tissue	mice	miR-21 miR-29b1 miR-29a	–	Hand et al. [130]
	Liver tissue	mice	miR222	–	Shen et al. [131]
	Liver tissue	human	miR-222	–	Dong et al. [132]
Cholestatic liver injuries	H69 and HIBEpiC (cell lines)	human	miR-221	–	Hu et al. [133]
	Liver tissue	Human rat	miR-200a miR-141 miR-200b miR-200c	miR-124	Xiao et al. [134]
Acute liver failure (ALF)	Liver tissue BNLCL2 (cell line)	mice	–	miR-1187	Yu et al. [135]
	Liver tissue BNLCL2 (cell line)	mice	miR-155 miR-125a/b miR-26b miR-15b miR-16 miR-21	miR-466f miR-467f miR-574 miR-93 miR-1187 miR-145 let-7b miR-329 miR-24	An et al. [136]
	Liver tissue HUH-7 (cell line)	human (adult and children)	miR-126 miR-130a miR-20a miR-520e miR-330 miR-150 let-7i miR-27a miR-494 miR-1224 miR-149	miR-503 miR-23a miR-663 miR-654 miR-152 miR-200b miR-183	Salehi et al. [137]
Non-alcoholic fatty liver disease (NAFLD)	Liver tissue HepG2 (cell line)	mice (liver) human (cell line)	–	miR-451	Hur et al. [138]
	Liver tissue HepG2 (cell line)	mice (liver) human (cell line)	miR-200a miR-200b miR-200c miR-146a miR-146b miR-152	–	Feng et al. [139]
Non-alcoholic steatohepatitis (NASH)	Liver tissue	mice and human	miR-21	–	Dattaroy et al. [140]
Viral hepatitis C	293T HepG2 HUH-7.5 (cell lines)	human	miR-122	–	Israelow et al. [141]

**Table 3:** Circulatory miRNAs differentially expressed in paediatric liver disease.

Disease	Sample Type	Sample Source	Method	Upregulated	Downregulated	References
Cystic fibrosis liver disease (CFLD)	Serum	Children	PCR array qRT-PCR	miR-122 (in CFLD) miR-21 and miR-25 (in CFnoLD)	–	Cook et al. [143]
Billiary atresia (BA)	Serum	Children	PCR array qRT-PCR	miR-200a miR-200b miR-429	–	Zahm et al. [144]
	Serum	Children	Microarray qRT-PCR	miR-92a-3p miR-4689 miR-150-3p	miR-4429	Dong et al. [145]
	Plasma	Children	NGS qRT-PCR	miR-200a-3p miR-574-5p miR-194-5p miR-432-5p miR-122-5p miR-100-5p miR-let7c-5p	miR-10b-5p miR-140-3p miR-26a-5p miR-126-3p miR-744-5p miR-370-3p miR-142-3p miR-23a-3p	Peng et al. [146]
Hepatitis B	Plasma	Children	PCR array qRT-PCR	miR-99a-5p miR-100-5p miR-122-5p miR-122-3p miR-192-5p miR-192-3p miR-194-5p miR-483-3p miR-855-5p miR-1247 miR-28-5p miR-30a-5p miR-30e-3p miR-125b-5p miR-193b-3p miR-215 miR-365a-3p miR-378a-3p miR-455-5p miR-455-3p miR-574-3p miR-let-7c	miR-654-3p	Winther et al. [147] Winther et al. [148] Winther et al. [149]

## Abbreviations

A	Adenine
AGO	Argonaute
ALD	Alcoholic liver disease
ALF	Acute liver failure
ALT	Alanine amino transferase
AST	Aspartate amino transferase
AUC	Area under the curve
BA	Biliary atresia
CF	Cystic fibrosis
CFLD	Cystic fibrosis-associated liver disease
CFnoLD	Cystic fibrosis no liver disease
CFTR	Cystic fibrosis transmembrane regulator
CHB	Chronic hepatitis B
eIF4	Eukaryotic translation-initiation factor 4
EMT	Epithelial to mesenchymal transition
ESCRT	Endosomal sorting complex required for transport
EV	Extracellular vesicle
HBeAg	Hepatitis Be antigen
HBsAg	Surface antigen of hepatitis B virus
HDL	High-density lipoprotein
Hh	Hedgehog
hnRNPA2B1	Heterogeneous nuclear ribonucleoprotein A2B1
HSC	Hepatic stellate cells
IRES	Internal ribosome entry site
miR	microRNA
miRNA	microRNA
mRNA	Messenger RNA
mRNP	Messenger ribonucleoprotein
MVE	Multivesicular endosomes
NAFLD	Non-alcoholic fatty liver disease
NASH	Non-alcoholic steatohepatitis
NGS	Next-generation sequencing
nt	Nucleotide
PABP	Poly(A) binding protein
pre-miRNAs	Precursor miRNAs
pri-miRNAs	Primary miRNAs
RISC	RNA-induced silencing complex
RNases	Ribonucleases
ROC	Receiver operating characteristic
SNP	Single nucleotide polymorphism
TGF-β	Transforming growth factor β
WHO	World Health Organization
